# Chemoenzymatic Site-Specific Labeling of Influenza Glycoproteins as a Tool to Observe Virus Budding in Real Time

**DOI:** 10.1371/journal.ppat.1002604

**Published:** 2012-03-22

**Authors:** Maximilian Wei-Lin Popp, Roos A. Karssemeijer, Hidde L. Ploegh

**Affiliations:** 1 Whitehead Institute for Biomedical Research, Cambridge, Massachusetts, United States of America; 2 Department of Biology, Massachusetts Institute of Technology, Cambridge, Massachusetts, United States of America; University of Wisconsin-Madison, United States of America

## Abstract

The influenza virus uses the hemagglutinin (HA) and neuraminidase (NA) glycoproteins to interact with and infect host cells. While biochemical and microscopic methods allow examination of the early steps in flu infection, the genesis of progeny virions has been more difficult to follow, mainly because of difficulties inherent in fluorescent labeling of flu proteins in a manner compatible with live cell imaging. We here apply sortagging as a chemoenzymatic approach to label genetically modified but infectious flu and track the flu glycoproteins during the course of infection. This method cleanly distinguishes influenza glycoproteins from host glycoproteins and so can be used to assess the behavior of HA or NA biochemically and to observe the flu glycoproteins directly by live cell imaging.

## Author Summary

Enveloped viruses such as the influenza virus cause significant disease in humans. In order to cause a productive infection, the virus particle must interact with the host cell using the viral proteins encoded within its genome. For many such viruses, it is possible to directly observe the early steps in infection, yet for technical reasons it has been extremely difficult to study the genesis of daughter virions as they bud off of infected host cells. Here we devised a chemoenzymatic labeling strategy to site-specifically append probes to the influenza hemagglutinin (HA) and neuraminidase (NA) proteins using the bacterial sortase A enzyme. Because labeling is confined to surface exposed HA and NA in the context of live, infected cells, it is possible to study budding biochemically and microscopically in real-time. Using this system, we can observe budding of flu virions from discrete sites at the cell surface. Our work will enable detailed investigation into the birth of viruses from infected host cells and can likely be applied to viruses other than influenza that have been similarly resistant to real-time microscopic observation during budding.

## Introduction

Enveloped viruses are composed of elements produced by and recruited from the infected cell. The formation of new virus particles occurs either on intracellular membranes or at the plasma membrane. The assembly of a nascent virion requires the coalescence of the envelope (glyco)proteins embedded in a proper lipid environment, and the recruitment of matrix and nucleocapsid proteins together with the viral genome [1]–[3]. How apposition of envelope components and viral genomes are controlled as a means of ensuring production of infectious progeny is not well understood.

The influenza virus particle contains a segmented, negative stranded RNA genome encoding 11 proteins, two of which the virus uses to interact with the host cell membrane [4]. Hemagglutinin (HA), a type I transmembrane protein, binds to sialoglycoconjugates on the surface of the host cell and mediates entry of the viral particle [5], [6]. HA also mediates fusion of the viral and host cell membranes to effectuate genome delivery to the cell to be infected [7]. Neuraminidase (NA), a type II membrane protein, is a sialidase that assists in release of virions from the infected cell [8].

The inability to label either flu HA or NA in a manner that allows continuous monitoring of surface disposition, surface distribution, and release has hampered the study of flu particle biogenesis. The use of antibodies, while feasible in principle, requires their introduction as fluorophore-conjugates that would crosslink viral proteins unless used as monovalent F(ab) fragments. Moreover, this labeling method is indirect. Studies that address particle biogenesis have also mostly used fixed cells and by design have not addressed virus release in real time. Visualization of the influenza glycoproteins in living cells demands a method for site-specifically modifying HA and NA, at the exclusion of all host proteins inserted into the very same membrane. We know of no successful attempts to achieve this by genetically tagging the flu glycoproteins with fluorescent proteins or with other methods that yield visible HA or NA by covalent modification in the context of an infectious virus.

We and others have developed a site-specific labeling method that exploits sortase transpeptidases found in gram positive bacteria [9], [10]. These enzymes cleave the five amino acid LPXTG recognition sequence between the threonine and glycine residues, forming an acyl-enzyme intermediate that is resolved by nucleophilic attack by the N-terminus of an oligoglycine peptide, forming a new amide bond. This reaction is portable: upon incubation with recombinant sortase A, proteins that carry an LPXTG motif are readily labeled with oligoglycine-based probes bearing a broad range of functionalities [11], [12]. The incoming nucleophile may carry any desired substituent for attachment, including fluorophores, biotin, lipids, or may even consist of other polypeptides- see [13], for review.

Here we report the creation of two influenza A/WSN/33 strains bearing the sortase cleavage site in the HA and NA proteins respectively. Infection of host cells with such strains allows site-specific labeling of HA or NA and allows us to observe the products of influenza infection in real time. We can thus visualize and examine biochemically the events that immediately precede viral release from the host cell surface, as well as the release of newly formed virus particles. The ability to execute sequential labeling reactions employing distinct tags allowed us to observe preferred sites from which virus particles are released.

## Results

### Generation of recombinant A/WSN/33 influenza viruses for sortase labeling

While purified virus particles can be labeled with lipophilic dyes, the dequenching of which reports on fusion of the incoming viral envelope with target endosomal membranes [14], [15], the production of new virions is more difficult to visualize. Neither flu neuraminidase nor hemagglutinin tolerate fusion to fluorescent proteins or other modules that allow site-specific covalent attachment of fluorophores—attempts to do so are incompatible with virus production and assembly. For N-terminal fusions to NA, this is likely the result of failure to insert into the ER during its biosynthesis. The bulky GFP moiety, when fused to the C-terminus of NA, likely compromises its functional activity and oligomerization. For HA, the only viable option would be to place GFP at the C-terminus, but fusions as small as a (His)_6_ epitope already impair virus assembly and fusogenic activity of HA [16]. Fusions to the N-terminus of mature HA have been reported [17], but with the caveat that such fusions undergo significant proteolysis and yield substantial amounts of wild-type HA protein. The inability to specifically label the flu glycoproteins for biochemical and visual observation has hampered an analysis of the virus budding process.

**Figure 1:**
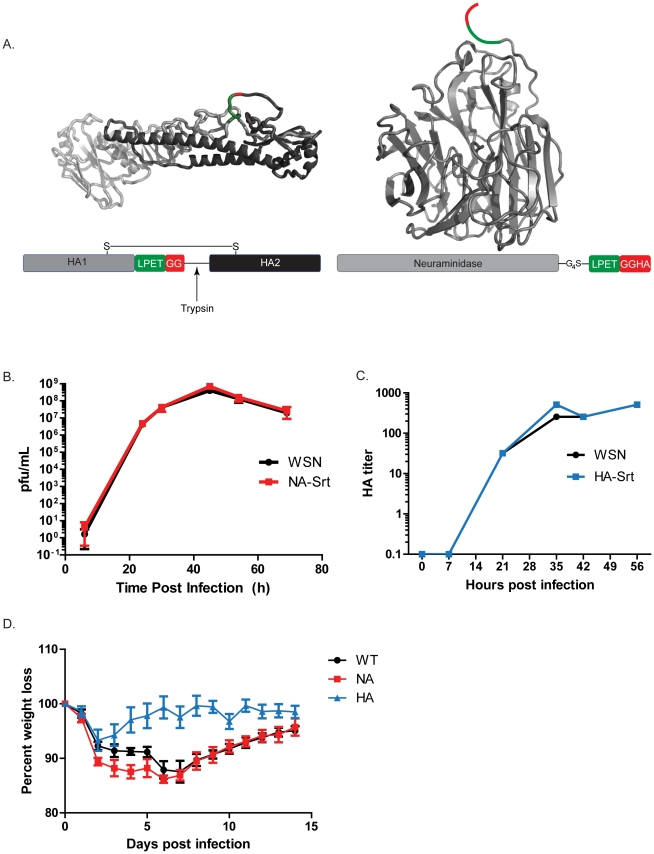
Generation of sortase compatible influenza viruses. (**A**) **Structural representation of sortase cleavage site mutations in hemagglutinin and neuraminidase.** Sortase cleavage sites including residues that remain with the labeled species (green) and those lost after sortase-medaited transpeptidation (red) were engineered into the indicated positions in hemagglutinin (left) and neuraminidase (right). Cartoons of crystal structures are depicted on top (PDB ID for HA: 1HA0, NA: 2HTY). (**B**) **NA-Srt multistep replication assay.** NA-Srt virus and wild-type WSN virus (n = 3) were used to infect MDBK monolayers at an MOI = 0.001 and viral supernatant was plaqued on MDCK cells at the indicated times. (**C**) **HA-Srt multistep replication assay** HA-Srt and wild-type WSN virus (n = 3) were used to infected MDCK monolayers at an MOI = 0.001 and viral supernatant was analyzed via standard hemaggutination assays. Shown are the last dilution factors at which hemagglutination of red blood cells still occurs. (**D**) **Infectivity of engineered viruses.** Mice (n = 4 in each group) were inoculated with 40000 pfu of the indicated virus and body weight was monitored at the indicated intervals. Shown is the mean and SEM.

We devised a sortase-based labeling method to overcome at least some of these limitations, and our findings are likely to be more generally applicable to other viruses with problematic labeling characteristics when relying on fusion with fluorescent proteins. The sortase labeling (sortagging) method is particularly well-suited for labeling of type II membrane proteins, as has been done for CD40L [11], CD74 and Dectin-1 [18] at the extracellularly exposed C-terminus. We installed an LPETG motif at the C-terminus of the A/WSN/33 NA protein, followed by an HA epitope tag (this epitope is absent from the A/WSN/33 hemagglutinin) (**Figure 1A**). Because the portion distal to the cleavage site is lost upon sortagging, the presence of the epitope tag allows monitoring of material not accessible to the enzyme in intact cells. We used the 12 plasmid reverse genetics system to generate recombinant A/WSN/33 flu particles bearing this sortaggable NA construct [19], and found that the resulting virus was infectious and indistinguishable from wild type virus in its ability to replicate in vitro (**Figure 1B**). We refer to this strain as NA-Srt.

HA is a type I membrane protein, synthesized as an HA0 precursor, which requires proteolytic cleavage by a trypsin-like activity to generate the disulfide-bonded HA1 and HA2 subunits. This cleavage exposes a key glycine residue at the N-terminus of HA2 that is essential for HA2 to retain its fusogenic activity [5]. We created a version of HA that allows trypsin cleavage in the loop that connects HA1 and HA2, with concomitant exposure of the sortase recognition sequence (placed immediately upstream of the trypsin cleavage site). Cleavage is likely to improve accessibility of the LPXTG motif, a requirement for efficient labeling [11], [20]. We therefore generated a recombinant A/WSN/33 flu strain bearing this sortaggable HA, and found that this strain, too, was not attenuated in vitro (**Figure 1C**). We refer to this strain as HA-Srt. The behavior of the NA-Srt virus was indistinguishable from that of the WSN parental strain when assessing virulence by monitoring weight loss in mice. Mice infected with sublethal doses of the HA-Srt virus also showed weight loss, albeit somewhat reduced compared to mice infected with wild-type virus. (**Figure 1D**). We conclude that the installation of a sortase tag on either NA or on HA does not seriously impair virus assembly, virus release and infectivity in vitro and in vivo.

### Sortase-mediated labeling of NA-Srt and HA-Srt virions and infected cell surfaces

**Figure 2:**
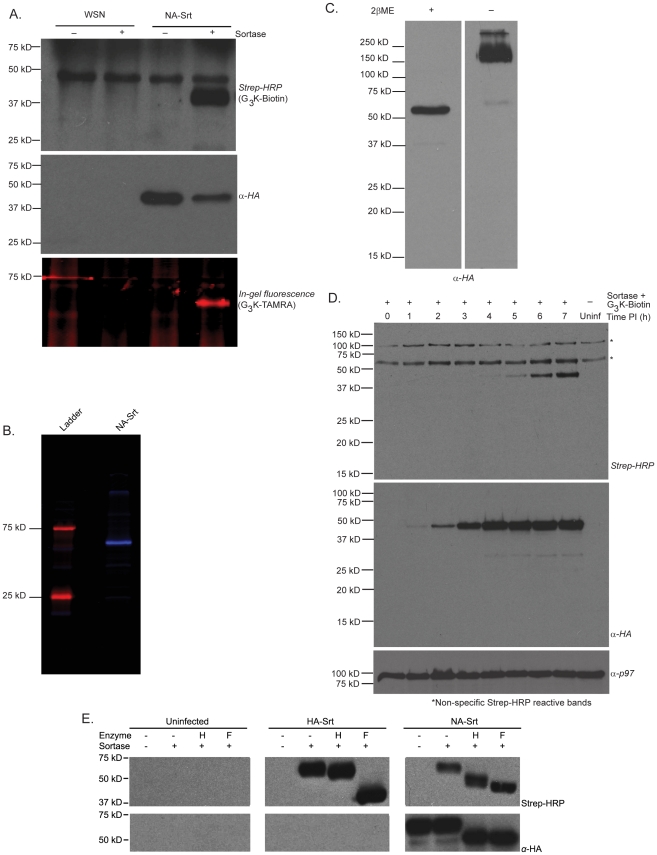
Engineered viruses are sortase substrates. (**A**) **NA-Srt virus is a sortase substrate.** NA-Srt virus bearing a sortase cleavage site appeneded to the C-terminus of neuramindase, followed by an HA epitope, was pelleted from tissue culture supernatant and incubated with 150 µM sortase A and 5 mM of the indicated probe at 37°C for 1 hour, with agitation. Streptavidin-HRP immunoblot (top panel) shows incorporation of a G_3_K(Biotin) probe with concomitant loss of the HA epitope (middle blot). A glycine based tetramethylrhodamine probe can also be incorporated (bottom panel, fluorescence gel). (**B**) **Highly purified NA-Srt virus is labeled with a glycine based AlexaFluor 647 probe.** NA-Srt virions were purified through a 20% sucrose cushion and further banded on a continuous 15–60% sucrose gradient before labeling with sortase (200 µM) and an AlexaFluor 647 probe (500 µM) for 2 hours at 37°C. The labeling reaction was fractionated by 12.5% SDS-PAGE and scanned by a Typhoon Imager. (**C**) **NA-Srt protein incorporated into virions is quantitatively forms disulfide bonded dimers.** NA-Srt virus was purified over a sucrose gradient and loaded on a 12.5% SDS-PAGE gel in the presence or absence of reducing agent (2-mercaptoethanol). Anti-HA epitope immunoblot displays the presence of the NA-Srt protein. (**D**) **Neuraminidase on infected MDCK cell surfaces is selectively labeled by sortase A.** MDCK cells were infected at MOI = 0.5 and at the indicated times post infection, were incubated for 30 min with 200 µM SrtA and 500 µM biotinylated nucleophile. Cells were collected, subjected to 12.5% SDS-PAGE, and immunoblots with streptavidin-HRP (top), anti-HA (middle), and anti-p97 (bottom, loading control) were performed. (**E**) **Glycosidase digestions of cell surface labeled HA-Srt protein.** MDCK cells were infected at an MOI of 0.4 overnight and labeled for 1 hour at 37°C with 100 µM sortase and 500 µM biotin probe. Cells were then lysed in glycoprotein denaturing buffer and digested with either PNGase F or EndoH, resolved by 12.5% SDS-PAGE, transferred to nitrocellulose, and used for immunoblotting with the indicated antibodies.

We obtained NA-Srt virions by sedimentation from the supernatants of infected MDCK cells, and subjected this material to sortagging with a biotinylated probe, followed by detection of biotinylated material by immunoblotting. Only in the presence of sortase and probe did we detect specific labeling, accompanied by the loss of the HA epitope tag, as expected. Based on the intensity of the HA-positive materials recovered, we estimate that the labeling efficiency of HA in the sedimented virus is approximately 70–80% for the conditions used (**Figure 2A**). This value is not atypical for sortase-mediated labeling reactions, which usually proceed to near-completion [11], [21]. Using sortase we similarly installed TAMRA or Alexa647 dyes on intact virions pelleted from tissue culture supernatant or further purified through a sucrose gradient (**Figure 2A** **and** **Figure 2B**). Dimerization of the NA-Srt protein incorporated into gradient-purified virions is unaffected by the LPETG tag (**Figure 2C**)

We infected MDCK cells with the NA-Srt virus and at different times post infection, we subjected cells to sortagging with a biotinylated triglycine-based probe (**Figure 2D**). We detected biotinylated, surface accessible NA by immunoblotting using streptavidin-HRP. We assayed for the unlabeled and cell-internal pools of NA by reactivity with an anti-HA epitope antibody. NA was first detectable at ∼1 hr post-infection and its levels peaked at ∼4 hrs, after which we observed no further increase. Sortase-mediated surface biotinylation of NA was detectable at 4 hrs post-infection and steadily increased over the duration of the experiment (7 hrs). Specificity of labeling is excellent: we observed no host cell proteins modified with the biotinylated probe.

Cells infected with the HA-Srt virus can be similarly labeled. Glycosidase digestions confirm that sortase labels only the cell surface pool of flu glycoproteins (**Figure 2E**), as follows. We labeled intact cells infected with either the HA-Srt or NA-Srt viruses with sortase using a biotinylated probe, followed by lysis and digestion with either Endoglycosidase H or PNGaseF. Immunoblotting showed that all of the biotinylated HA-Srt protein is partially EndoH-resistant, indicating successful traversal of the secretory pathway. Because mature NA and HA carry both complex-type and high mannose-type oligosaccharides [22], [23], resistance to digestion with EndoH is always partial, as seen by comparison with the PNGaseF digestion product. The entire unlabeled, anti-HA reactive pool of NA-Srt protein was fully EndoH-sensitive, however, as evident from a comparison with the PNGaseF digestion products. The fraction of NA-Srt inaccessible to sortagging is thus indeed composed of cell-internal NA-Srt protein. We conclude that only the cell surface pool of influenza glycoproteins, poised for incorporation into nascent virions, is labeled upon incubation of infected cells with sortase and a suitable probe.

### Biochemical study of flu release from infected cells

Having established the specificity of labeling of the sortagging method, we examined the biogenesis of flu virions and their release from infected cells through biochemical analysis and by live cell imaging. We labeled flu HA-Srt protein on the surface of infected and metabolically labeled cells by exposure to trypsin, followed by sortase-mediated installation of a single biotin at the C-terminus of HA1. We performed a pulse-chase experiment to examine the kinetics of arrival of HA-Srt protein at the cell surface, and its subsequent release from the infected cell as assembled virions. We did not ascertain the presence of all subgenomic RNA fragments in the material released from the infected cell, as we have no means of testing whether individual particles carry a full complement of subgenomic RNAs, or whether the released materials contain substantial amounts of defective particles with incomplete sets of RNAs. However, our data are consistent with the release of HA-Srt into the medium corresponding to assembly and release of progeny virions (see below).

**Figure 3:**
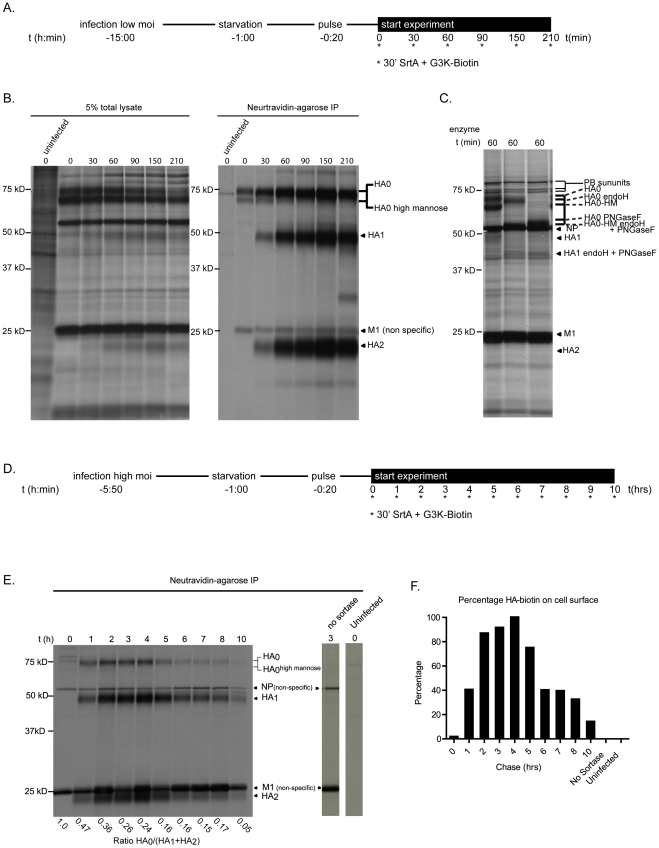
Surface distribution of HA-Srt measured via pulse-chase analysis. (**A**) **Experimental setup.** Confluent layers of MDCK cells were infected at an MOI of 0.05 for 13 hrs, starved for 60 minutes and labeled with [^S^35]Cysteine/Methionine for 20 minutes. Cell surface accessible HA-Srt was labeled for 30 minutes with 100 µM sortase and 250 µM biotin probe at the indicated timepoints. (**B**) **Pulse-chase analysis of virus budding shortly after labeling.** Cells were lysed in 0.5% NP-40 lysis buffer and biotin labeled HA-Srt was recovered via immunoprecipitation on streptavidin-agarose for 3 hrs. A 5% aliquot of the total cell lysate was removed prior to the immunoprecipitation (left panel). Bound complexes were eluted from beads with 2× SDS sample buffer, resolved by 12.5% SDS-PAGE and visualized via autoradiography (right panel). (**C**) **Glycosidase treatment of the cell lysate.** A fraction of the total cell lysate at the 90 minute timepoint was denatured in glycoprotein denaturing buffer and digested with either PNGase F or EndoH. Proteins were resolved by 12.5% SDS-PAGE and visualized via autoradiography. (**D**) **Experimental setup.** Confluent layers of MDCK cells were infected with an MOI = 0.5 for 4.5 hrs, starved for 60 minutes and labeled with [^S^35] Cysteine/Methionine for 20 minutes. Cell surface accessible HA-Srt was labeled for 30 minutes with 100 µM sortase and 250 µM biotin probe at the indicated timepoints. (**E**) **Surface behavior of HA-Srt analyzed via pulse chase analysis.** MDCK cells were lysed at the indicated timepoints and analyzed as in 3B. Cells infected with HA-Srt for 3 h but not treated with sortase and uninfected cells were similarly analyzed (right two lanes). Densitometric quantification of radioactivity was performed to calculate the ratio of HA0 over HA1 and HA2. (**F**) **Quantification of surface HA-Srt** Densitometric quantification of radioactivity was performed to quantify the total levels of HA-Srt at the cell surface. The amount at t = 4 hrs was set at 100% to and other levels compared in…

We initiated metabolic labeling with ^35^S labeled Cysteine/Methionine at ∼5 hours post-infection, a time when robust viral protein synthesis is ongoing. For some experiments, we infected cells at a low multiplicity of infection (MOI) (**Figure 3A**), and started metabolic labeling at 14 hrs post-infection, when most of the cells are infected by progeny HA-Srt virus produced by the cells infected initially. In this setting, we observe a similar if not greater amount of labeling than for cells infected at a high MOI (**Figure 3D**), indicating that the LPETG-tag is neither lost nor interferes with virus replication (**compare** **Figure 3B** **and** **Figure 3E**). During the chase, we performed sortase labeling for 30 minutes at the indicated time points. We lysed the ^35^S-labeled sortagged cells and subjected them to affinity purification on a neutravidin-agarose matrix to recover the biotin-modified HA1 and associated proteins (**Figure 3B**). At the 0 min time point of this experiment (it includes a 30 minute incubation with sortase, during which intracellular transport of glycoproteins continues), no labeled HA1 is recovered, indicating that the newly synthesized pool of HA requires at least 30 minutes to reach the cell surface. We observe a steady increase in surface exposed (sortase accessible) HA-Srt protein as well as a minor fraction of associated, uncleaved HA0. We attribute the presence of this HA0 to incomplete cleavage of the HA trimer by trypsin added to the medium. In this manner we recover -as part of a trimer- some HA0 devoid of biotin, along with the sortase modified, biotinylated HA. As expected, we do not observe biotinylated HA0 by streptavidin blot (data not shown). We do observe a small amount of HA0 at the cell surface at the 0 min timepoint. This we attribute to a minor portion of labeled HA1 not detectable via autoradiography. The HA0 recovered at early time points is composed of both the mature HA0 and the high mannose intermediate (**Figure 3C**). As expected, HA1 and HA2 are recovered together because of their covalent association, which persists after cleavage of HA0. We examined the behavior of HA-Srt protein at later chase times (**Figure 3E** **and** **Figure 3F**) and again observed an increase in labeling, after which the amount of labeled HA-Srt protein decreases. By 10 hours of chase, less than 20% of the material that successfully reached the cell surface and is labeled by sortase is retrieved from the cells, indicating that most, but not all HA labeled in the course of the pulse is released from the cell, presumably as intact virions. As infected cells show clear signs of cytopathic effects at late time points post-infection, cellular functions required for virion assembly are likely to be compromised, thus preventing complete release of all viral products, a situation that likely applies in vivo as well.

**Figure 4:**
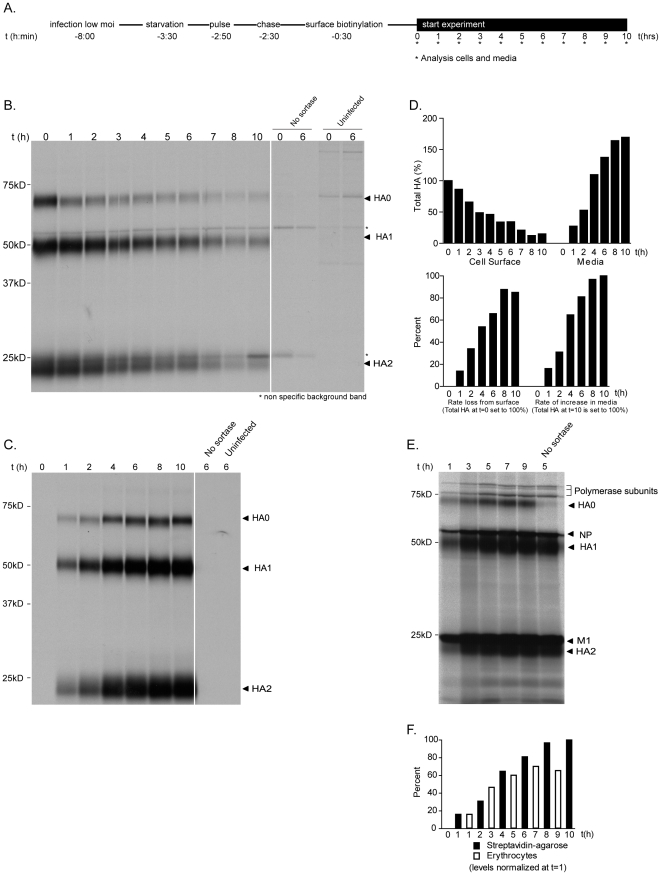
Site-specifically labeled HA-Srt protein is incorporated into virions. (**A**) **Experimental setup.** Confluent monolayers of MDCK cells were infected with an MOI = 0.5 during 4.5 hours after which cells were starved and pulse-labeled with [^S^35]Cysteine/Methionine for 20 minutes. After a 2 hour chase, a second pulse-labeling was performed using 100 µM sortase and 250 µM biotin probe to label surface accessible HA-Srt. At indicated timepoints, both cell supernatant as well as cell lysate was analyzed for presence of viral proteins. (**B**) **Surface behavior HA on infected MDCK cells analyzed via affinity adsorption to neutravidin-agarose.** At indicated timepoint, cells were lysed in 0.5% NP40 buffer and biotin labeled HA-Srt remaining at the cell surface recovered via affinity adsorption on neutravidin-agarose. Proteins were eluted with 2× SDS sample buffer, resolved by 12.5% SDS-PAGE and visualized via autoradiography. (**C**) **Accumulation of HA-biotin in supernatant analyzed by affinity adsorption to neutravidin-agarose.** Accumulation of biotin-HA-Srt in the supernatant of the cells analyzed in 4B was measured via immunoprecipitation on neutravidin-agarose. Supernatant was lysed via addition of NP40 buffer prior to biding to beads. Proteins were eluted with 2× SDS sample buffer, resolved by 12.5% SDS-PAGE and visualized via autoradiography. (**D**) **Quantification of HA loss from the cell surface.** Densitometric quantification of radioactivity was performed on autoradiographs from figure 4B and 4C. Total levels of HA-Srt were quantified relative to the levels at the cell surface at t = 0 hrs (top graph). To quantify the kinetics of budding, loss of HA-Srt from the cell surface was quantified as percent reduction relative to the t = 0 timepoint at the cell surface. The rate of accumulation in the cell supernatant was quantified relative to the maximal amount recovered at the t = 10 hrs timepoint. (**E**) **Accumulation of whole virus particles analyzed by affinity adsorption to chicken erythrocytes.** Accumulation of complete virus particles in the cell supernatant was measured via affinity adsorption on chicken erythrocytes. Supernatant from cells analyzed in 4B was removed at indicated timepoints and mixed with chicken erythrocytes for 30 minutes at 4°C. Cells and bound viral particles were lysed in 2× SDS sample buffer, proteins resolved on 12.5% SDS-PAGE and visualized via autoradiography. (**F**) **Kinetcs of virus accumulation as analyzed by adsorption to neutravidin-agarose versus erythrocytes.** Densitometric quantification of radioactivity was performed to compare the rate of HA-Srt accumulation in the supernatant compared to whole viral particles (4C versus 4E). Numbers were normalized at t = 1 hrs at which both methods were used.

To determine the fate of surface-labeled HA-Srt, we subjected infected cells to metabolic ^35^S –Met/Cys pulse labeling, followed by a chase period of 2 hours to allow radioactive HA-Srt protein to accumulate on the cell surface (**Figure 4A**). We labeled intact infected cells with sortase A and a biotinylated probe and recovered biotinylated HA-Srt protein from cell lysates as well as from the media (**Figure 4B** **and** **Figure 4C**). We observe a gradual increase in HA-Srt released into the media over time, corresponding to the rate of loss of biotinylated HA-Srt from the cell surface (**Figure 4D** **bottom panel**). However, we detect more released HA-Srt protein than is accounted for by the loss from the cell surface (**Figure 4D** **, top panel**). We attribute this difference to the fact that biotinylated HA-Srt is tightly associated with non-biotinylated HA-Srt in intact virus particles, which are retrieved by the neutravidin-agarose matrix along with the sortagged fraction. Metabolically labeled HA-Srt does not bind non-specifically to this matrix, as virus-infected cells exposed to the biotinylated probe in the absence of added sortase do not show any signal.

Does the decrease in cell-associated, biotinylated HA1, accounted for by the appearance of biotinylated HA-Srt protein in the supernatant, correspond to the release of virus particles? To demonstrate this, we recovered from tissue culture supernatants radiolabeled viral proteins not modified by sortase through adsorption of released virions onto chicken erythrocytes (**Figure 4E**), and visualized the adsorbed materials by SDS-PAGE and autoradiography. Because binding of the virions to erythrocytes occurs via HA, any other protein recovered by low speed sedimentation of erythrocytes must be part of an adsorbed virus particle. Indeed, we detect the other viral proteins upon suitable exposure of the autoradiograms. These include polypeptides with the assigned molecular masses of the RNA polymerase subunits, as well as NP and M1, all of them in quantities proportional to their methionine/cysteine content and to the reported copy numbers in intact virions [24]. We next compared the kinetics of HA accumulation in the media for the fractions recovered via Neutravidin-agarose or on chicken erythrocytes. Levels of total HA at the 1 hr time point were quantified and all other time points were normalized to these values (**Figure 4F**). We observe indistinguishable kinetics for HA-Srt accumulation in the media, underscoring our conclusion that biotinylation does not affect budding of HA-Srt.

### Visualization of influenza glycoproteins in living, infected cells

Having established the specificity of the labeling reaction and the ability of the labeled flu glycoproteins to be incorporated into virions and released into the culture supernatant, we next visualized virus budding and release by labeling biotinylated, surface exposed HA-Srt protein with streptavidin-modified quantum dots. To examine the behavior of surface disposed HA-Srt and its release from infected cells using a similarly modifiable control protein as a reference, we generated an MDCK cell line stably transduced with CD154/CD40L, equipped with a sortase tag [11]. Like the NA-Srt and HA-Srt proteins, this molecule is readily labeled with biotin using sortase, yet should not be actively incorporated into nascent virions and so allows for a direct comparison with flu HA-Srt.

**Figure 5:**
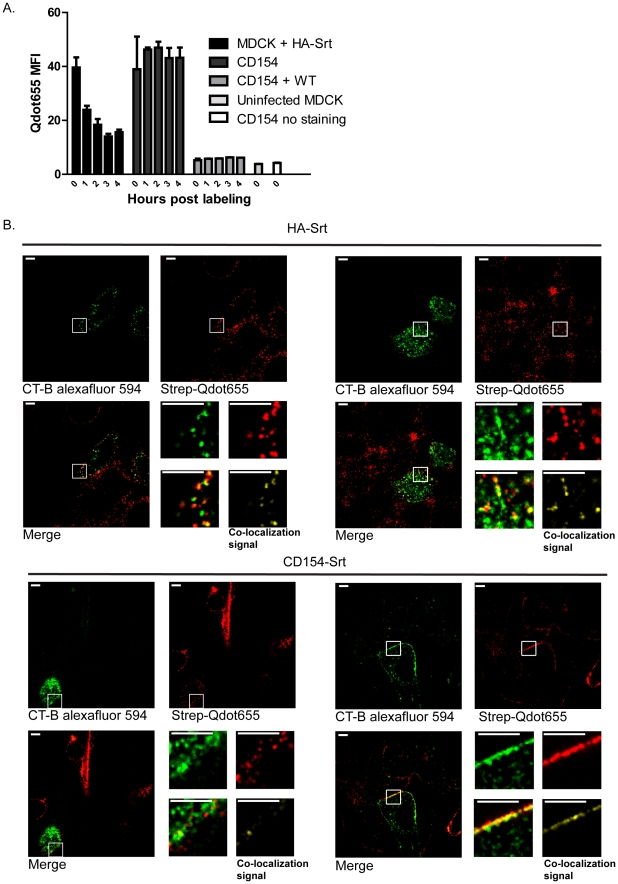
Visualization of HA-Srt protein by site-specific sortase labeling followed by streptavidin-Qdot staining. (**A**) **Budding of Qdot labeled virus analyzed via flow cytometry.** Confluent MDCK cells or CD154/CD40L expressing MDCK cells were infected with HA-Srt or Wild-type virus at an MOI = 1 for 4 hrs. Cell surface accessible HA-Srt or CD154 was labeled for 30 minutes with biotin using 100 µM sortase and 250 µM biotin probe. Biotin labeled surface molecules were further labeled via a 5 minute incubation with 20 nM Qdot655 or 10 nM Qdot655 in the case of uninfected CD154/CD40L expressing MDCK cells. Total fluorescent intensity of the cells was measure via flow cytometry at the indicated timepoints. (**B**) **HA-Srt colocalizes with lipid rafts at the cell surface.** Confluent layers of MDCK cells were infected at an MOI of 0.35–0.5 for 4.5 hours. Cell surface molecules of infected MDCK cells or CD154/CD40L expressing MDCK cells were labeled with 100 µM sortase and 250 µM biotin probe for 30 minutes. Biotin labeled HA-Srt of CD154 were further labeled with 20 nm and 10 nm Qdot655 respectively and lipid rafts were stained with 20 µg/ml CT-B conjugated to Alexa fluor 594. Images were acquired immediately after staining. Scale bars = 5 µm.

Incubation of MDCK cells that display biotinylated HA-Srt or CD154 were readily labeled with quantum dots. We examined the fluorescence intensity as a function of time after labeling by cytofluorimetry of infected, labeled cells (**Figure 5A**). Whereas the levels of fluorescence recorded for labeled CD154/CD40L were constant, those for labeled HA-Srt decreased exponentially over the first few hours of incubation. When we infected labeled CD154/CD40L-expressing cells with wild type WSN virus, we also observed constant staining intensity but at a lower level, presumably because host protein synthesis was much reduced in the virus-infected cells. Labeled CD154/CD40L is obviously not incorporated into budding virions.

We labeled HA-Srt infected MDCK cells grown on glass coverslips with sortase A and biotinylated oligoglycine probe, followed by staining with streptavidin-functionalized quantum dots. Cells prepared in this manner were directly imaged by spinning disc confocal microscopy to observe the behavior of the HA-Srt protein during the course of virus production and release. The influenza glycoproteins are known to be inserted into host cell membrane regions referred to as lipid rafts, operationally defined as insoluble in non-ionic detergents. One key component of lipid rafts is the ganglioside, GM1, the levels of which oscillate with cell cycle status [25]. We co-stained HA-Srt labeled cells with fluorescently labeled cholera toxin (CTx), a marker for GM1, and observed variable CTx staining between MDCK cells, presumably because of asynchronous growth and rapid internalization of CTx [20]. Uninfected MDCK cells show similar heterogeneous CTx staining, indicating that virus infection is not the cause of this variability (data not shown). We observe co-localization of CTx with patches of quantum dot staining in HA-Srt infected cells (**Figure 5B**), but in CD40L/CD154 labeled cells, quantum dot staining shows only partial overlap with CTx staining, reflecting broad distribution of labeled CD154 in the cell membrane (**Figure 5B**). Where there are patches of quantum dot-stained HA-Srt, we see evidence of CTx colocalization, while for quantum dot labeled CD154, this is not always the case. Of note, cells that do not stain with CTx nonetheless label perfectly well with streptavidin-modified Qdots (and hence correspond to flu-infected cells) in a pattern that is indistinguishable from that seen in the adjacent, CTx positive cells. Although GM1 is a raft component, the organization of the plasma membrane apparently does not require its presence in a CTx-reactive form for the organization of HA-Srt.

**Figure 6:**
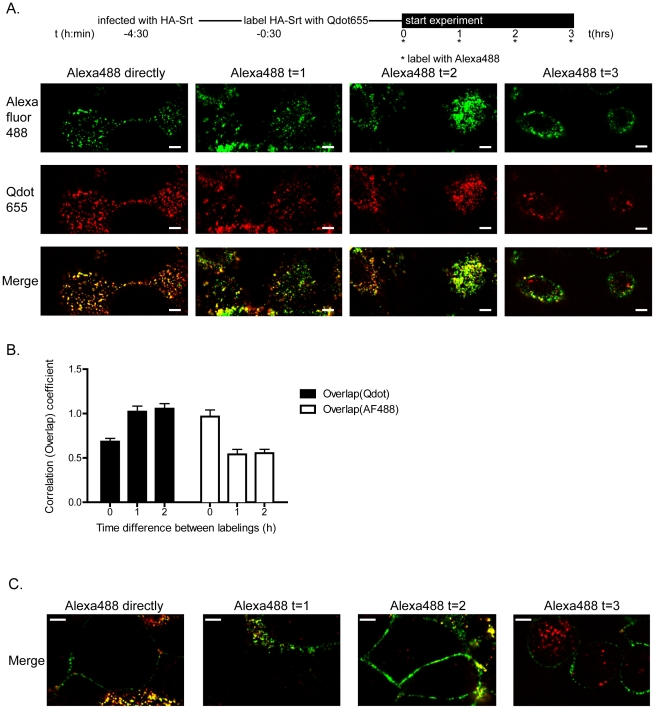
Visualization of HA-Srt behavior by site-specific pulse labeling using sortase A. (**A**) **Time course of surface appearance of HA-Srt protein.** Experimental setup is depicted on top. MDCK cells were infected with HA-Srt and labeled with 250 µM biotin probe using 100 µM sortase followed by labeling with Qdot655. At indicated timepoints after the initial labeling, a second round of labeling was performed using sortase and Alexafluor 488 probe for 60 minutes at 4°C. Images were acquired immediately after labeling. Scale bars = 5 µm. (**B**) **Overlap coefficients.** The overlap coefficients for Qdot655 signal with the AF488 signal and the AF488 signal with the Qdot655 signal are displayed for the images shown in figure 6A as well as the images from Supporting Fig. S1 (n = 10). (**C**) **Intracellular appearance of Qdot655 conjugated HA-Srt.** Spinning disc confocal microscopy images were taken of the samples from figure 6A (stained with AF488 and Qdot655) at a focal plane midway through the cells in order to visualize both plasma-membrane as well as the intracellular space, where Qdots accumulate at later timepoints (t = 3 h). Scale bars = 5 µm.

We conducted a pulse labeling experiment using sortase to determine the fate of HA-Srt protein labeled with biotin and streptavidin modified Qdots, followed by a second round of site-specific labeling with an oligoglycine based Alexa fluor 488 probe [18] to distinguish this second pool of labeled material from the first round of labeled HA-Srt, at a later stage of maturation and virus budding. Infected cells were first labeled with sortase and a biotinylated probe, followed by staining with streptavidin quantum dots (HA-Qdot). After 30 minutes, this first pulse was followed by a second round of labeling using sortase A and an oligoglycine-Alexa 488 probe (HA-488) at 4°C to inhibit endocytosis of free dye (**Figure 6A** **, Figure S1**). When labeling with the Alexa488 probe is initiated directly after quantum dot staining, we observe clear colocalization of the Qdot signal with the Alexa488 signal for most of the patches, and this is reflected as an increase in the overlap coefficient of the Qdot signal with the Alexa488 signal over time (**Figure 6A** **and** **Figure 6B** **, Supporting Figure S1**). Given the near quantitative labeling we observe for HA at the cell surface, the Alexa488-labeled pool of HA must therefore correspond to HA molecules inserted at sites where HA, labeled with Qdots in the first round of sortagging, has coalesced. These insertion sites appear as discrete dots. When Alexa 488 staining is initiated at later time points, HA-488 is located not only in previously established HA-Qdot patches, but we observe the presence of an increasing number of new Alexa488 spots of HA-Srt outside of the Qdot patches. This results in a decrease in the overlap coefficient of the HA-488 signal with the Qdot signal over time (**Figure 6B**).

The majority of HA-Qdot remains co-localized with HA-488 over the course of the experiment (**Figure 6B**), suggesting that new HA-Srt is continuously being exported to the same membrane patches during budding. However, while colocalization with HA488 persists, both the number of HA-Qdot patches as well as their intensity decreases over time when compared to HA-488 (**Figure 6A** **, Figure S1**). Although it is a formal possibility that budding may not have been completed during the 2–3 hour interval, these observations may also suggest that patches of HA-Srt on the cell surface serve as sites of multiple budding events. At the 3 hr timepoint, little of the initial HA-Qdot signal remains, paralleling exactly the decrease observed by flow cytometry (**Figure 5A**). A substantial HA-Qdot signal remains and is found inside cells instead of at the cell surface at later timepoints (**Figure 6C**). The release of HA-Qdot tagged virus in the confined environment of this tissue culture experiment unavoidably leads to adsorption and internalization of labeled virus by adjacent, uninfected as well as onto already infected cells. Labeling with Alexa-488 probe increases at every time point, consistent with continued output of HA-Srt.

**Figure 7:**
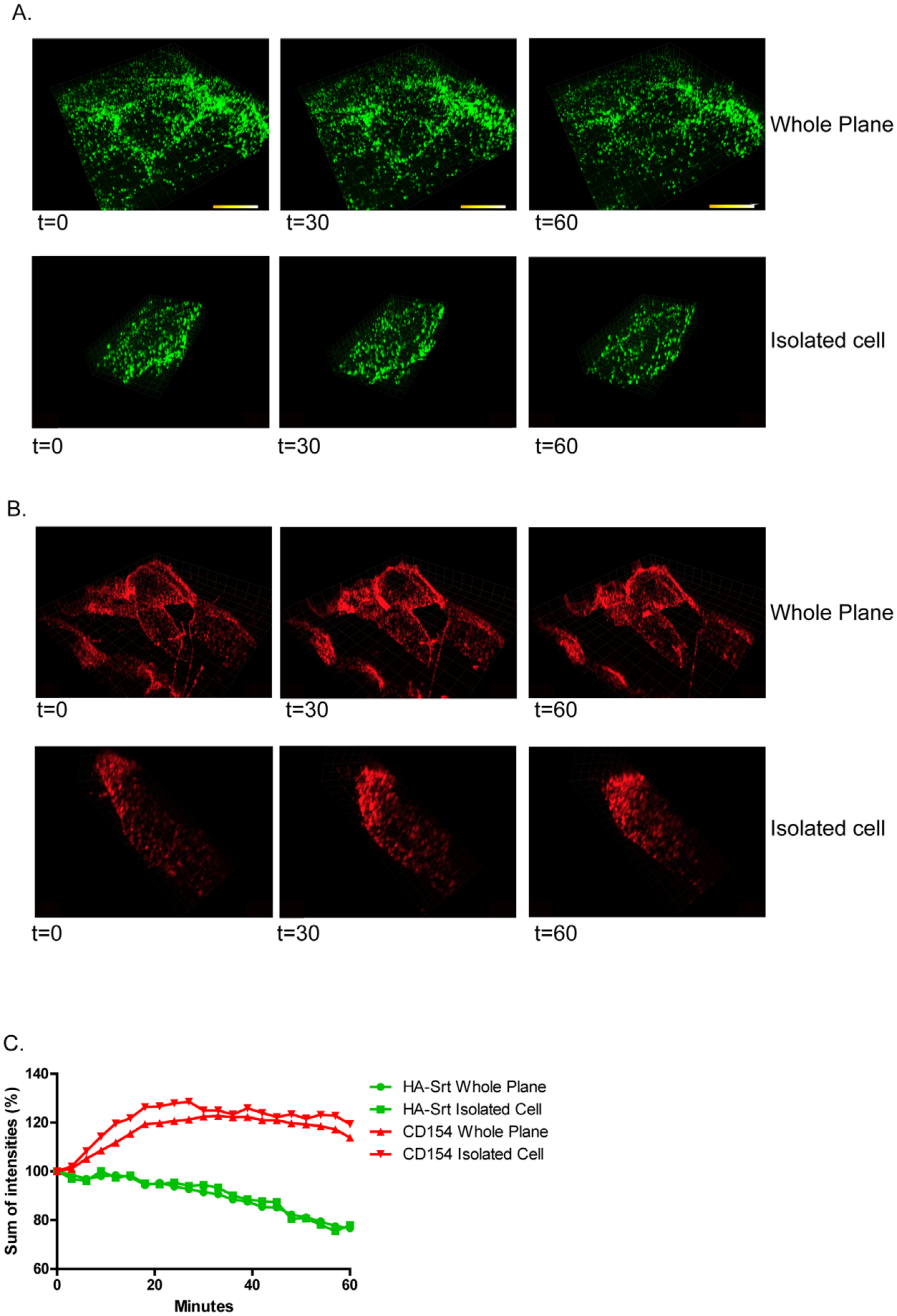
3-D reconstruction of HA-Srt dynamics on the cell surface. (**A**) **Real time imaging of the disappearance of HA-strep-Qdot655 from the cell surface.** MDCK cells were infected with HA-Srt, labeled with 100 µM sortase and 250 µM biotin probe followed by 20 nmQdot655 as described. Images were acquired every three minutes in Z-stack series of 8 µm in 21 images. Imaris software was used to create 3D images. Top figure displays the entire frame while the bottom image is focused on a single cell. Scale bars = 5 µm. (**B**) **Real time imaging of CD154/CD40L on the cell surface.** CD154/CD40L expressing MDCK cells were labeled with 100 µM sortase and 250 µM biotin probe and subsequently with 10 nm Qdot 655. Images were acquired every three minutes in Z-stack series of 8 µm in 21 images. Imaris software was used to create 3D images. Top figure displays the entire frame while the bottom image is focused on a single cell. Scale bars = 5 µm. (**C**) **Sum of pixel intensities in 7A and 7B.** The sum of pixel intensities at every timepoint was analyzed with imaris software and percent reductions quantified relative to the first timepoint.

We next studied the behavior of HA-Srt at the cell surface in real time by timelapse imaging (**Figure 7A**). We labeled HA-Srt with biotin probe and streptavidin-Qdots at 4 hours post infection and acquired images over the following 60 minutes. We see a clear disappearance of HA-Qdot from the infected cells over this time course (**Figure 7A**). We do not see this loss of Qdot signal in CD154/CD40L-expressing cells labeled in the same fashion (**Figure 7B**). This loss of Qdot signal is apparent when the total sum of pixel intensities is plotted (**Figure 7C**). The initial increase seen in CD154/CD40L control cells is likely due to a slight movement of the cells which cannot be restricted in our system and indicates that the decrease in HA-Qdot signal seen must be a minimum estimate of HA-Qdot release. We also observe an increase in clustering of the Qdot patches over time in the CD154/CD40L control cells. This may also account for the increase in signal intensity, as clustering of quantum dots is known to increase the on-time of blinking Qdots [26] Timelapse movies show a similar decrease in Qdot signal for the infected cells relative to the CD154/CD40L-expressing control cells (data not shown). We detect both an apparent decrease in the total number of patches as well as a decrease in the intensity. This is in agreement with the observations made by flow cytometry (**Figure 5A**) and the timecourse of pulse labeling with sortase (**Figure 6A** **, Figure S1**).

## Discussion

For viruses such as vaccinia virus, which tolerates GFP extensions in many of the proteins it encodes, direct visualization of viral replication has provided major insights into the interactions between host cell and pathogen [27]. For viruses such as flu that are refractory to labeling with fluorescent proteins, other methods are urgently needed. We here devised a system to observe the behavior of influenza glycoproteins in cells infected with a fully functional virus. Our approach leverages the sortase labeling technique through the generation of influenza viruses that carry the short sortase recognition sequences in their glycoproteins, resulting in minimally modified viral gene products in the context of an infectious particle. Upon infection of tissue culture cells, the engineered viruses show behavior identical to the parental strain in terms of infectivity, replication kinetics, and viral protein synthesis. Observation of the sortase-labeled glycoproteins thus reflects very well the behavior of their wild-type counterparts. By combining this labeling approach with live-cell imaging, we can monitor the behavior of the influenza glycoproteins in real time. We observe extensive colocalization of surface-disposed flu glycoproteins with lipid rafts, as inferred from staining with CTx. In MDCK cells, flu HA and NA are enriched in lipid rafts based on their transient insolubility in cold TX100 and on colocalization with CTx [28].

Several aspects deserve emphasis. First, the specificity of the reaction and its reliance on an active enzyme limit all labeling to proteins that bear the sortase recognition sequence. We have not detected spurious incorporation of label into proteins not specifically designed to serve as sortase substrates. This applies not only to our earlier results with CD154/CD40L [11], dectin-1 and CD74 [18], but is extended here to flu NA and HA. The requirement for proteolytic conversion of HA0 into HA1 and HA2 suggested the possibility of installing a sortase motif upstream of the trypsin cleavage site without affecting the folding or fusogenic activity of the HA1-HA2 heterodimer, and our results validate this approach. Second, the glycosidase digestions performed on HA and NA unequivocally demonstrate the selectivity of the sortase reaction for fully mature surface-disposed proteins. In this regard, the method compares favorably in terms of ease and specificity with other chemical (iodination, biotinylation) or enzymatic (lactoperoxidase-catalyzed iodination) surface labeling methods, whose products require additional purification steps to enable analysis of the protein(s) of interest. Third, the size of the substituents introduced is minimal compared to that of a fluorescent protein such as eGFP or other enzymatically active modules used for site-specific covalent modification [29]. Given the failure to construct infectious flu when incorporating GFP into any of its structural proteins including NA and HA, the small size of our probes and the choices of label available have enabled, for the first time, the visualization of flu release from the surface of infected cells in real time. Fourth, it is possible to perform sequential labeling experiments and install labels that distinguish between each pool of labeled protein, and so independently monitor each pool of labeled product. This allowed us to generate a starting population of HA-positive cells labeled with quantum dots, from which we generated, at set intervals, a second population of cells with a distinct label installed on HA. Using this approach we demonstrate that HA-Srt, labeled in a first round of sortagging, identifies patches into which HA, tagged in a subsequent round of labeling, is inserted. Although it is possible that budding may not have been completed within this time interval (2–3 hours), an alternative interpretation is the existence of specialized sites that serve as a platform for coalescence of viral glycoproteins (a typical flu virion requires some 400 HA and 30 NA molecules). Whether the identity of such patches corresponds to lipid rafts or specializations of lipid rafts is not clear, but the ability to visualize such sites opens the possibility of identifying host factors that control the construction of these sites as launching pads for new virions. We anticipate that this system will yield a robust method to visualize the kinetics of particle formation and, in combination with perturbations of host cells, will reveal host proteins that contribute to the process of influenza virion biogenesis.

## Materials and Methods

### Ethics statement

All animal protocols were conducted in accordance with the Guide for the Care and Use of Laboratory Animals of the National Institutes of Health. All animals were maintained according to the guidelines of the MIT Committee on Animal Care (CAC). These studies were approved by the MIT CAC (protocol #1011-123-14). All infections were performed under avertin anesthesia, and all efforts were made to minimize suffering.

### Protein production and peptide probe synthesis

Sortase was produced as described (Popp et al., 2007). Peptide probes were produced as described [18].

### Generation of recombinant viruses

Mutant viruses were generated by reverse genetics using plasmids as described [19]. The hemagglutinin and neuraminidase plasmids were modified by standard molecular biology techniques to carry the sortase cleavage site. All viruses, including the wild-type WSN virus used were rescued as described [19]. Nucleotide and protein sequences for modified portions of flu glycoproteins are below.

### Protein and nucleotide sequence of HA WT (WSN)

Protein Sequence (Trypsin cleavage site is in bold)

…CPKYVRSTKLRMVTGLRNIPSIQY**RG**LFGAIAGFIEGGWTGMIDGWYGYHHQNEQGSGYAA…

Nucleotide Sequence (Trypsin cleavage site is in bold)

…tgcccaaaatatgtcaggagtaccaaattgaggatggttacaggactaagaaacatcccatccattcaatac**agaggt**ctatttggagccattgctggttttattgaggggggatggactggaatgatagatggatggtatggttatcatcatcagaatgaacagggatcaggctatgcagcg…

### Protein and nucleotide sequence of HA-Srt

Protein Sequence (Sortase recognition site is in italics, trypsin cleavage site is in bold)

…CPKYVRSTKLRMVTGLRNIPSIQY*LPETGG* **RG**LFGAIAGFIEGGWTGMIDGWYGYHHQNEQGSGYAA…

Nucleotide Sequence (Sortase recognition site is in italics, trypsin cleavage site is in bold)

…tgcccaaaatatgtcaggagtaccaaattgaggatggttacaggactaagaaacatcccatccattcaatac*ctgcccgagaccggcggc* **agaggt**ctatttggagccattgctggttttattgaggggggatggactggaatgatagatggatggtatggttatcatcatcagaatgaacagggatcaggctatgcagcg…

### Protein and nucleotide sequence of NA WT (WSN)

Protein Sequence

…SGSIISFCGVNGDTVDWSWPDGAELPFTIDK-

Nucleotide Sequence

agtgggagcatcatttctttttgtggtgtgaatggtgatactgtagattggtcttggccagacggtgctgagttgccgttcaccattgacaagtag

### Protein and nucleotide sequence of NA-Srt

Protein Sequence (Sortase recognition site is in italics, HA epitope is in bold)

…SGSIISFCGVNGDTVDWSWPDGAELPFTIDKGGGGS*LPETGG* **YPYDVPDYA-**

Nucleotide Sequence (Sortase recognition site is in italics, HA epitope is in bold)

agtgggagcatcatttctttttgtggtgtgaatggtgatactgtagattggtcttggccagacggtgctgagctcccgttcaccattgacaagggcgggggcggatcccttcctgaaactggtgga**tacccatacgatgttccagattacgct**tag

### Stable transduction of MDCK cells

CD154/CD40L bearing an LPETG tag [11] was cloned into pLHCX and used to make retrovirus as described [30]. Retrovirus was used to infect MDCK cells as described [30] and cells were selected in 250 µg/ml Hygromycin B.

### Viral assays

Viral titer was assessed by plaque assay on MDCK cells as described [28]. For multi-step replication assays, MDBK cells were infected at an MOI of 0.001 and incubated for the indicated times in viral growth medium (VGM, DME with 0.3% BSA) supplemented with 0.5 µg/ml TPCK-treated trypsin (NA-Srt) or 1 µg/ml TPCK treated trypsin (HA-Srt). For NA-Srt multi-step replication, 100 µl of media was plaqued at the indicated time points on MDCK cells grown with 0.5 µg/ml TPCK-trypsin. For HA-Srt, HA-Srt and wild-type WSN virus (n = 3) were used to infected MDCK monolayers at an MOI = 0.001 and viral supernatant was analyzed via standard hemagglutination assays.

### Viral particle purification

Viral particles from tissue culture supernatant were concentrated by pelleting through a 20% sucrose cushion (Sigma) at 25000 rpm in an SW-28 rotor for 120 minutes. Where indicated, virus was further purified through a continuous 15%–60% sucrose gradient, centrifuged at 30,200 rpm in an SW-40.1 rotor for 3 hours.

### Sortase labeling of virions

For biotin and TAMRA labeling, virions were pelleted from tissue culture supernatant without a sucrose cushion. The pellet was resuspended in 1× sortase buffer and labeled with 150 µM sortase A/5 mM probe for 1 hour at 37°C. For Alexa647 probe labeling, sucrose gradient purified virions were mixed with 200 µM sortase A and 500 µM probe at 37°C for 2 hours.

### Sortase labeling of live cell surfaces for immunoblot

MDCK cells were plated in a 24 well dish at 70% confluency the night before the experiment. Cells were infected at an MOI of 1 and 0,1,2,3,4,5,6 or 7 hours post infection, cells were incubated with 100 mM sortase A and 100 mM G_5_K-biotin probe [11] in VGM for 30 minutes at 37°C. Cells were washed extensively in PBS, collected, and lysed in 1% SDS with protease inhibitor cocktail (Roche). A BCA assay was performed (Pierce) and 20 µg of lysate was loaded for western blotting.

### Mouse infections

Mice (n = 4 in each group) were inoculated intranasally with 40000 pfu of the indicated virus and body weight was monitored at the indicated intervals. Balb/C mice and B6129SF2/J mice were purchased from the Jackson Laboratory (stock# 000651 and 101045 resp). Mice were anesthetized with Avertin and infected intranasally with 40.000 PFU WT, HA-Srt or NA-Srt virus. Infection was followed by daily monitoring of weight loss and animals were euthanized with C0_2_ when weight loss exceeded 20% of initial body weight.

### Cell surface labeling and glycosidase digestion

MDCK cells were infected at an MOI of 0.4 overnight and labeled for 1 hour at 37°C with 100 mM sortase and 500 mM biotin probe. Cells were then lysed in glycoprotein denaturing buffer (New England Biolabs) and total protein in lysates were quantiatated by BCA assay (Pierce). Five micrograms of cell lysate was digested with either PNGase F or EndoH according to manufacturer's directions (New England Biolabs), resolved by 12.5% SDS-PAGE, transferred to nitrocellulose, and used for western blotting with the indicated antibodies.

### Pulse chase experiments

MDCK cells were grown in 6-well tissue culture dishes and infected with HA-Srt at either an MOI 0.05 for 14 hrs or MOI 0.5 for 4.5 hrs as indicated in figure legends. Cells were starved with methionine- and cysteine-free DMEM for 45 minutes at 37°C followed by a 20 minute pulse labeling with [^S^35]Cysteine/Methionine (perkin elmer) at 0.77 mCi/ml in methionine- and cysteine-free DMEM. Chase was initiated by addition of VGM supplemented with 1 mM methionine, 0.2 mM cysteine and 1 µg/ml TPCK treated trypsin. At indicated timepoints during chase, cell surface HA molecules were labeled with 0.25 mM G_3_K-Biotin and 0.1 mM Srt_Aureus_ for 30 minutes at 37°.

#### Analysis of cell lysate

Cells were washed 3 times in PBS and lysed in NP40 lysis buffer (0.5%NP40, 150 mM NaCl, 5 mM MgCl_2_, 25 mM Tris pH 7.4). Immunoadsportions were performed with 35 µl Neutravidin (Thermo Scientific) agarose for 2 hrs at 4°C after an inital preclear of 4–10 hrs using 120 µl Immobilized protein A beads (Repligen). Immune complexes were eluted by boiling in reducing sample buffer, subjected to 10% SDS-PAGE and visualized by autoradiography.

#### Analysis of cell supernatant

For analysis of virus proteins in supernatant, cells were infected and pulse labeled as described above and subsequently incubated with 1 ml VGM supplemented with 1 µg/ml trypsin-TPCK at 37°C. At indicated timepoints, supernatant was collected and residual cells removed by short centrifugation. For immunoadsorption, virus was lysed by addition of NP40 lysis buffer to a final concentration of 0.5% NP40. Biotin labeled HA molecules were recovered via immunoadsorption on 35 µl neutravidin after a 4 hr preclear with 40 µl immobilized protein A beads. Immunecomplexes were eluted by boiling in reducing sample buffer, subjected to 10% SDS-PAGE and visualized by autoradiography.

For analysis of total levels of radiolabeled virus, cell supernatant was incubated for 1 hr with 8^E^6 chicken red blood cells at 4°C under gentile agitation. Erythrocytes and bound virus were collected via centrifugation, lysis in NP40 lysis buffer containing DNAse and further lysed by bioling and scraping in reducing sample buffer. Viral proteins were visualized by autoradiography after 10% SDS-PAGE.

Densitometric quantification of radioactivity was performed on a PhosphorImager (Fujifilm BAS-2500) using Image Reader BAS-2500 V1.8 software (Fujifilm) and Multi Gauge V2.2 (Fujifilm) software for analysis.

### Analysis of Qdot labeled virus via flow cytometry

MDCK cells or CD154 control cells were cultured o/n in 24-well tissueculture dishes. Cells were infected with HA-Srt or WT virus at an MOI of 1. At 4 hrs post-infection, cell surface molecules were labeled by addition of 0.1 mM Srt_aureus_ and 0.25 mM G_3_K-Biotin for 30 minutes at 37°C. Cells were washed in PBS and incubated for 5 minutes with either 20 nM qdot655 (MDCK cells and CD154 cells infected with WT-WSN) or 10 nM qdot655 (CD154 cells). Cells were incubated at 37°C and at indicated timepoints collected via addition of trypsin and kept on ice. Cells were analyzed immediately on a FACSCalibur flow cytometer (BD Biosciences) and FlowJo software.

### Confocal microscopy

MDCK cells or CD154/CD40L expressing MDCK cells were cultured in 35 mm glass bottom dishes (MatTek Corporation) and infected with HA-Srt or WT virus at an MOI of 0.35–0.5 for 4 hours. Cell surface molecules bearing the LPETG motif were labeled by incubation with 0.1 mM SrtA_aureus_ and 0.25 mM G_3_K-biotin for 30 minutes at 37°C. Cells were washed and incubated with 20 nM (MDCK) or 10 nM (CD154) qdot655 (Invitrogen) for 5 minutes. After extensive washing, cells were incubated with VGM containing 1 µg/ml trypsin-TPCK. For surface labeling with (G)_3_K-Alexafluor 488 probe, cells were incubated with 0.1 mM SrtA_aureus_ and 20 nM probe for 1 hr at 4°C to inhibit non specific endocytosis of free dye. For staining of lipid rafts, cells were stained with 20 µg/ml Alexa fluor 594 conjugated choleratoxinB dye (invitrogen) for 5 minutes at room temperature immediately following Qdot labeling and imaging performed directly after.

Images were acquired using an Andor Revolution spinning disk system with Yokogawa CSU-X1 spinning disk head, Andor iXon+ EM-CCD camera, 488 nm diode laser for excitation, emission discrimination with an emission filterwheel, Piezo Z100 z-stage on a Nikon Ti-E motorized microscope stand with a 100× 1.49NA Plan Apochromatic objective all controlled with the Andor iQ2 software (version 2.0). Temperature, CO2 and humidity was controlled with a LiveCell stage-top incubation system (Pathology Devices).

Analysis was performed using Imaris, Volocity and ImageJ software. For colocalization analysis, background correction was applied using ImageJ background correction with a rolling ball radius of 20 pixels. Images were further analyzed with Volocity colocalization analysis software. Background threshold was manually set using a background ROI to correct side effects of possibly remaining background pixels.

Imaris software was used for analysis of timecourse Z-stack series. Brightness of images was adjusted using the linear stretch algorithm with the maximum set to 55. Background was corrected for using a 17 µm filter width. Imaris software was used to create 3D images as well as the quantification of pixel intensities.

## Supporting Information

Figure S1 **Additional Visualization of HA-Srt behavior by site-specific pulse labeling using sortase A.** Cells were processed as described in Fig. 6A. Merged images of the Qdot 655 (red) and Alexafluor 488 (green) signal are shown. Processed areas are outlined in white. Scale bars = 5 µm.
(TIF)
