# Supplementary figures and images for: Chemoenzymatic Site-Specific Labeling of Influenza Glycoproteins as a Tool to Observe Virus Budding in Real Time
Source: PLoS Pathog. 2012 Mar 22;8(3):e1002604. doi: 10.1371/journal.ppat.1002604 (PMC3310791; doi:10.1371/journal.ppat.1002604)

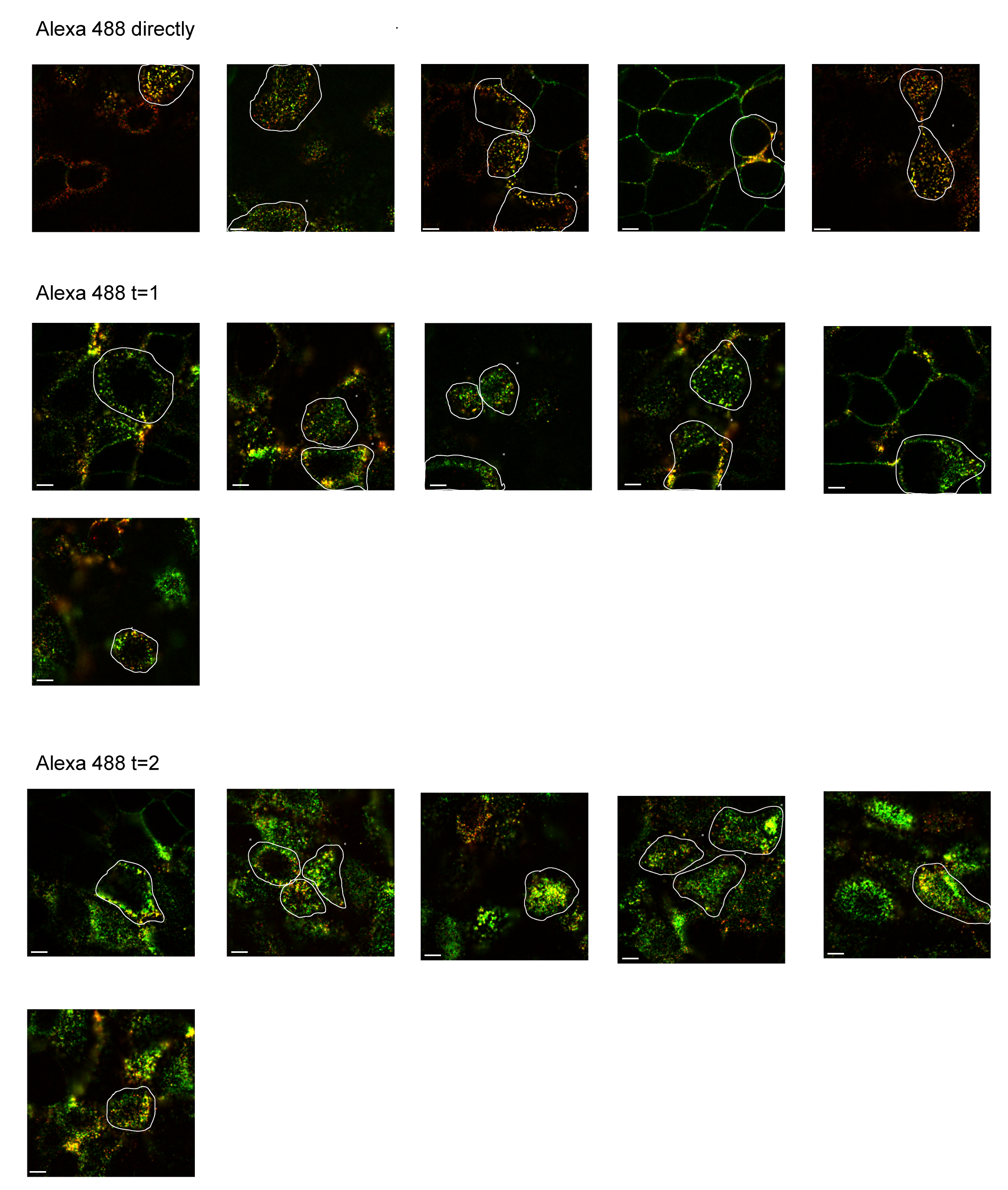

Supplement: Figure S1 — Additional Visualization of HA-Srt behavior by site-specific pulse labeling using sortase A. Cells were processed as described in Fig. 6A. Merged images of the Qdot 655 (red) and Alexafluor 488 (green) signal are shown. Processed areas are outlined in white. Scale bars = 5 µm. (TIF) [file ppat.1002604.s001.tif]
